# Hemophagocytic Lymphohistiocytosis and Gastrointestinal Bleeding: What a Surgeon Should Know

**DOI:** 10.1155/2015/745848

**Published:** 2015-06-25

**Authors:** S. Popeskou, M. Gavillet, N. Demartines, D. Christoforidis

**Affiliations:** ^1^Department of Surgery, Lugano Regional Hospital, Switzerland; ^2^Department of Hematology, University Hospital of Lausanne, Switzerland; ^3^Department of Surgery, University Hospital of Lausanne, Switzerland; ^4^University Hospital of Lausanne, Switzerland

## Abstract

This paper presents to the surgical community an unusual and often ignored cause of gastrointestinal bleeding. Hemophagocytic syndrome or hemophagocytic lymphohistiocytosis (HLH) is a rare medical entity characterized by phagocytosis of red blood cells, leucocytes, platelets, and their precursors in the bone marrow by activated macrophages. When intestinal bleeding is present, the management is very challenging with extremely high mortality rates. Early diagnosis and treatment seem to be the most important factors for a successful outcome. We present two cases and review another 18 from the literature.

## 1. Introduction

Gastrointestinal (GI) bleeding is a common cause for hospital admission to surgical wards. In westernized countries, the annual incidence of upper GI bleeding is approximately 100 −200/100,000 and for lower GI bleeding 20–30/100,000. The estimated mortality ranges from 6% to 10% for upper GI bleeding and from 2% to 4% for lower GI bleeding [1–4]. Most episodes of lower GI bleeding resolve spontaneously, while others can be treated endoscopically or by interventional radiology. A minority of patients become hemodynamically unstable and require emergency surgery. In such cases, the source of bleeding is not always clear and the surgeon may face the difficult decision to perform extensive colonic resections, at a price of significant mortality rates (up to 25%), without the certitude of controlling the source of bleeding [5–7]. The most frequent causes of lower GI bleeding are diverticulosis and arterial-venous malformations. Other frequent causes are colitis, neoplasms, inflammatory bowel disease, and hemorrhoids [8, 9].

In this paper, we report the dramatic cases of two patients who presented massive lower GI bleeding originating from diffuse GI mucosal ulcerations secondary to acute Epstein Barr virus (EBV) proliferation and hemophagocytic lymphohistiocytosis (HLH) and review the literature for GI bleeding associated with this rare syndrome.

## 2. Case 1

A 26-year-old male patient, treated for Crohn's disease with azathioprine (150 mg/day) for 3 years, was admitted to the ENT department of a community hospital for treatment of a febrile pharyngitis with 10-day cefuroxime. Lab tests revealed leucopenia (2.7 g/L, 89% neutrophils, 8% monocytes) and a mild elevation of the liver enzymes. Clinically and radiologically, splenomegaly was present. An active EBV infection (IgG and IgM positive) was diagnosed on hospital day 7 and treatment with valacyclovir (1 gr × 3/day) was started. At the same day, the patient presented massive lower GI bleeding requiring admission to the ICU. The initial emergency colonoscopy revealed severe pancolitis with multiple ulcerations which was attributed to a flare of Crohn's colitis. Three days later (day 10), despite multiple transfusions, he became hemodynamically unstable due to continuous bleeding (CRP 121 mg/L, procalcitonin 1.21 mcg/L). The patient was therefore transferred to our University Hospital. Upon arrival, he developed a hypovolemic shock requiring massive blood transfusions and an emergent total colectomy with ileostomy was performed. Broad-spectrum antibiotherapy with piperacillin/tazobactam (4.5 gr × 4/day) and prednisone 50 mg/d was initiated in the immediate postoperative period. A thoracic CT-scan showed bilateral pulmonary infiltrates, which were attributed to transfusion related acute lung injury (TRALI). Blood tests revealed pancytopenia and increasing levels of ferritin (2600 mg/L) thought to be due to the combination of inflammation and azathioprine treatment. Three days later (day 13), the patient was transferred back to the ICU of the first hospital.

There, severe mononucleosis with persistent high EBV viremia despite antiviral treatment was diagnosed. The patient presented multiple episodes of upper GI bleeding that required further repeated transfusions. Upper endoscopy revealed hemorrhagic gastroduodenitis with multiple bleeding ulcers. Blood tests revealed a further increase of ferritin (up to 15,000 *μ*g/L) and LDH (up to 4000 U/L) together with worsening pancytopenia and finally agranulocytosis. Repeated bone morrow biopsies finally revealed HLH with macrophage activation syndrome and hemophagocytosis. The patient was treated with high doses of steroids alone and exhibited transient improvement but eventually expired following multiple organ failure 12 days later. The biopsies from gastric, duodenal, and colonic tissues all revealed an EBV-associated lymphoid proliferation. The autopsy concluded to a generalized EBV infection with lymphoid proliferation of the entire digestive tube and multiple organs, with associated secondary macrophage activation syndrome.

## 3. Case 2

A 61-year-old male patient in good general health presented with an episode of significant lower GI bleeding. The patient had had a minor similar episode 4 months ago. One month ago, he had felt fatigue and repeated episodes of fever without any other specific symptoms.

Upon primary admission to another hospital, the patient was tachycardic and slightly hypotensive but improved after massive hydration and was transferred to our center. An upper endoscopy revealed only a small Mallory-Weiss type ulcer, which seemed unlikely to have been the source of bleeding. An angio-CT-scan of the abdomen revealed extensive diverticulosis with signs of transverse colitis but without a precise source of bleeding. The patient was treated conservatively but, in the following days, he developed fever and an important inflammatory syndrome (CRP 253 mg/L, normal leucocyte count) with rhabdomyolysis, acute renal failure, and high levels of LDH (1451 U/L) and ferritin (1200 mg/L) along with anemia and progressive thrombocytopenia. Three days after his admission, he once again became hemodynamically unstable due to recurrent massive low GI bleeding. From the time of patient's entry to that point, he had received a total of 10 units of RBC. Emergency laparotomy with intraoperative colonoscopy and enteroscopy was performed. Findings were an important inflammatory status around the transverse colon, a small intestinal perforation at the distal ileum, and 10–20 ulcerated inflammatory lesions in the ileum, one of which was actively bleeding. A resection of 60 cm of ileum was carried out and the patient was admitted to the ICU for postoperative surveillance. Histopathology of the ileum revealed a high grade NK-T lymphoma Epstein Barr virus (EBV) positive.

**Figure 1:**
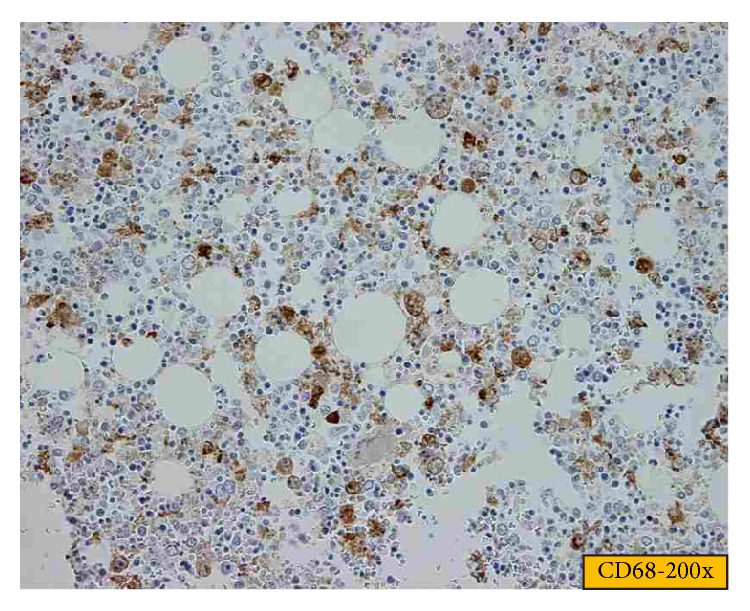
Hemophagocytosis in the bone marrow of Case 2.

Two days after surgery, the patient became septic. A thoracoabdominal CT-scan showed bilateral pleural effusions but no specific septic source. In the absence of radiological signs that would explain the patient's septic status, a “second look” laparotomy was performed but no source of sepsis was identified. Subsequently, the patient's condition deteriorated rapidly. He developed disseminated intravascular coagulation and finally a new episode of GI bleeding. Based on the above listed observations together with ferritin levels at 15000 mcg/L, HLH was suspected and a treatment with CHOP regimen (cyclophosphamide 750 mg/m^2^ day 1, doxorubicin 50 mg/m^2^/day 1, Oncovin 1.4 mg/m^2^/day 1, and prednisone 60 mg/m^2^/day 1 to 5) plus etoposide was initiated. No bone marrow biopsies were performed as death due to hemodynamic instability followed the next day. Autopsy revealed a NK-T extranasal EBV + lymphoma with intestinal localization. Bone marrow presented with massive hemophagocytosis without any evidence of tumor infiltration (see Figure 1).

## 4. Discussion

Hemophagocytic lymphohistiocytosis (HLH) or hemophagocytic syndrome represents a rare pathophysiologic entity characterized by a hyperinflammatory state, cytokine deregulation, impaired function of cytotoxic T-cells (CTL), and natural killer (NK) cells in addition to hemophagocytosis (i.e., phagocytosis by activated macrophages of red blood cells, leucocytes, platelets and their precursors in the blood marrow, spleen, or other lymphoid organs) [23, 24].

There is an inherited or familial form (FHLH) and an acquired or secondary HLH [25]. The FHLH incidence is estimated to be 0.12/100.000 children per year and has its onset under the age of 1 year in the majority (70%–80%) of patients [26]. Patients with inherited immune deficiencies like the Chediak-Higashi syndrome as well as the X-linked proliferative syndrome are also at high risk to develop HLH.

Secondary HLH may occur at all ages [24] and is usually diagnosed in association with malignant (mostly with cutaneous or anaplastic lymphomas), autoimmune, or infectious diseases. Infection associated with HLH is mostly associated with viral infections of the herpes group, in particular EBV and cytomegalovirus (CMV) [23, 25]. EBV induced HLH may mimic or favor T-cell lymphoma and other EBV-linked lymphoproliferative disorders [23]. Other nonviral agents such as bacteria, mycobacteria, protozoa, and fungi can more rarely trigger HLH [24]. Cases of HLH after immunization with measles vaccine, probably in predisposed patients, have also been documented [27].

The dominant clinical characteristics of HLH are persistent fever, usually unresponsive to antibiotics, splenomegaly and/or hepatomegaly, lymphadenopathies, icterus, rash, anorexia, and GI bleeding. Neurological symptoms, such as seizures or cranial nerve palsies, are rare in the acquired form but are found in up to 1/3 of patients with FHLH [25, 28–30].

Laboratory findings include cytopenia, in at least two cell lineages, elevated hemolysis parameters, hypofibrinogenemia (and possibly disseminated intravascular coagulation), hypertriglyceridemia, and hyperferritinemia. Inflammatory markers like the C-reactive protein may be quite high due to the syndrome's inflammatory state. Special markers include increased soluble CD25 (alpha-chain of the interleukin (IL-2) receptor) reflecting increased T lymphocytes activation and turnover and profoundly decreased or absent NK cell activity [24, 29, 31]. The Th1/Th2 biomarker is another recently developed diagnostic tool [32].

Diagnosis in patients with clinical suspicion of HLH requires bone marrow aspiration and biopsy. Positive biopsies reveal normal maturation of all cell lineages, normal or increased cellularity, and infiltration with macrophages/histiocytes associated with hemophagocytosis [25]. However, hemophagocytosis is often initially absent, requiring a repeated exam [24]. Due to the insidious nature of HLH, diagnosis is difficult, needs to be based on a combination of clinical and biological criteria, and is frequently established after the patient's death [22, 25, 33].

Infections must be actively sought as they may be the trigger of the syndrome and represent a treatable cause. Patients should be tested for EBV, CMV, herpes simplex, and adenovirus [24]. Screening for less frequent infectious agents, inherited immune deficiencies, and underlying malignancies should be done according to clinical suspicion.

HLH, whether acquired or familial, is a severe condition and, if left untreated, can lead to rapid deterioration and death. There is no high level evidence to guide treatment; the most widely accepted strategy is aggressive systemic therapy with various combinations of immunosuppressive and chemotherapeutic agents, along with the treatment of the underlying causes, if identified. For the severe forms of the FHLH, hematopoietic stem cell transplantation is currently considered to be the only curative option [32]. FHLH therapies with antithymocyte globulin, steroids, cyclosporin A, and intrathecal methotrexate to pediatric patients have been reported with 73% rapid and complete response [30]. Due to the lack of studies in the adult population, patients are usually treated with a regimen adapted from pediatric protocols [29]. For adult patients with HLH secondary to pathogens other than EBV, supportive care and treatment of the underlying infection are associated with recovery in 60%–70% [34]. EBV-associated HLH is almost universally fatal if untreated, with death resulting usually from multiorgan failure [31, 35].

The goal of this paper is to raise awareness among surgeons about the rare medical entity of HLH, which may present as a highly life threatening form of GI bleeding. In both cases presented in this paper, the surgeon was faced with a clinical picture of severe GI bleeding. These patients had presented a series of systemic clinical and laboratory findings compatible with HLH (fever unresponsive to antibiotics, splenomegaly, EBV infection, cytopenia, and high ferritin levels) earlier in the course of their illness, but diagnosis of HLH was not suspected until late. Surgical therapy for the GI bleeding was challenging in both cases as the bleeding source had not been localized precisely preoperatively. In the first patient, total colectomy was performed, but bleeding recurred, possibly from the documented coexisting gastroduodenal ulcerations. In the second patient, intraoperative colonoscopy and enteroscopy helped localize the source and guide the extent of the resection, but bleeding recurred as well. The role of surgery cannot be denied in the acute setting of life threatening GI bleeding. However, in these cases, earlier diagnosis and medical treatment of HLH might have prevented fatal recurrence.

The true incidence of GI bleeding in association with HLH is hard to estimate. Guo et al., in a retrospective analysis of 41 children with HLH, reported a rate of 12.2% [36]. In our review of the literature on HLH with associated GI bleeding, we found only a few other case reports (Table 1). Similar to our two cases, mortality was very high (12 deaths out of 18) and almost always secondary to uncontrollable GI bleeding. Of course, the severity of bleeding in this small review may be overrated due to selection and publication bias. The origin of bleeding can be localized throughout the GI tract. Our two patients bled form the colon, the small bowel, and the stomach. The bleeding source can be diffuse or from linear isolated ulcers. These gastrointestinal lesions are secondary to transmural lymphohistiocytic infiltration of macrophages as reported in histological findings [22], which in addition to bleeding may lead to perforation, as in one of our patients. Septic complications may be masked from the underlying hyperinflammatory state caused by HLH. Emergent surgery for intestinal resections or embolization was part of successful treatment in some reports [15, 20, 36]; in others, similar to our patients, it did not help control bleeding definitively [11, 19, 20, 22]. Administration of recombinant activated factor VII seemed to provide short temporary but no definitive control of bleeding in some reports [22, 37].

Hemophagocytic lymphohistiocytosis (HLH) is an elusive medical entity, difficult to diagnose. It may be present as life threatening GI bleeding and pose a challenging clinical problem to the surgeon. Associated cytopenia, high ferritin, and low fibrinogen levels, in combination with symptoms and signs such as fever, hepatosplenomegaly, skin rash, and lymphadenopathies, should raise suspicion early. Viral (EBV, CMV, and herpes simplex virus) and other (*Mycobacterium tuberculosis* and* Mycoplasma pneumonia*) infections should be looked for as they may represent treatable causes. Treatment requires a multidisciplinary approach and surgery may provide a temporary solution for GI bleeding or perforation, but early diagnosis and systemic therapy are the only hope for cure.

## Figures and Tables

**Table 1:** 

Author	*n*	Age	Concomitant systemic disease	Predominant symptoms and signs	Treatment (surgical/medical)	Outcome
Kanaji et al. [10]	1	25	—	Fever, abdominal pain, and lower GI bleeding	Methylprednisolone (1000 mg/day for 3 consecutive days), g-globulin (0.5 g/day for 3 consecutive days), broad-spectrum antibiotics (PAPM 1 g/day), gabexate mesilate (2500 mg/day), ulinastatin (20 × 10^4^ U/day), and citicoline (1 g/day).	Survived

Eguchi [11]	1	44	Lupus nephritis	Melena, colonic perforation, and peritonitis	CMV immunoglobulin, methyl-prednisolone (1 g/day for three days), prednisolone (60 mg/day), and ganciclovir (dose not specified). Colonoscopic clipping and embolisation of a bleeding sigmoid ulcer	Died

Bhagwati et al. [12]	1	55	—	Fever, abdominal pain, GI bleeding, and acute renal failure	Prednisolone (30 mg/day) and amphotericin and ganciclovir (doses not specified). Multiple transfusions	Died

Koketsu et al. [13]	1	35	—	Lower GI bleeding and fulminant “ulcerative colitis”	Methylprednisolone (1,000 mg/day for 3 consecutive days), subtotal colectomy, and ileostomy with rectal preservation	Survived

Nakase et al. [14]	2	68	—	Massive GI bleeding	https://www.jstage.jst.go.jp/browse/rinketsu/31/2/_contents?	Died
		80	—	Loss of consciousness and MOF	https://www.jstage.jst.go.jp/browse/rinketsu/31/2/_contents?	Died

Ina et al. [15]	1	76	—	Abdominal pain, massive GI bleeding, and jejunal ulcers	Jejunal resection (30 cm) after per operatory endoscopy	Survived

Yashima et al. [16]	1	63	Aplastic anemia	Massive GI bleeding and MOF	Methylprednisolone pulse therapy	Died

Wu et al. [17]	1	23	Hepatitis A and hepatitis C	Fever, jaundice, and massive GI bleeding	Bibliotheque	Died

Takai et al. [18]	1	20	—	Fever, hepatosplenomegaly, and massive GI bleeding	https://www.jstage.jst.go.jp/browse/rinketsu/31/2/_contents?	Died

Hayakawa et al. [19]	1	73	Polyarteritis nodosa	Massive GI bleeding	Methylprednisolone pulse therapy, intravenous immunoglobulin (5,000 mg for 3 days), and weekly intravenous VP-16 (200 mg/day). Embolisation of ileum artery branches	Died

N'Guyen et al. [20]	4	32.2 ± 3.7	Human cytomegalovirus infection present in all 4 patients	Fever, cytopenia, hyperferritinemia, hypertriglyceridemia, and GI bleeding; one patient with pericarditis and tamponade, one with colitis, and one with 2 necrotic gastric ulcers	Corticoids and antiviral therapy (ganciclovir and/or valganciclovir) + anti-TNF	Survived
					Corticoids and antiviral therapy (ganciclovir and/or valganciclovir) + anti-TNF	Survived
					Corticoids and antiviral therapy (ganciclovir and/or valganciclovir) + total colectomy	Survived
					Corticoids and antiviral therapy (ganciclovir and/or valganciclovir). Emergency surgery for 2 gastric ulcer perforations (operation not specified)	Died

Tunç and Ayata [21]	1	4	Visceral leishmaniasis	High fever, malaise, fatigue, massive GI bleeding, and opportunistic infections	Immunoglobulin (IVIG) (400 mg/kg for 5 days), antibiotics (ceftazidime (100 mg/kg/24 h) and amikacin sulfate (15 mg/kg/24 h)), AmBisome (3 mg/kg/day for 5 days), and methylprednisolone (30 mg/kg/day) for 3 consecutive days	Died

Celkan et al. [22]	2	1.5	—	Submandibular lymphadenopathy and hepatomegaly massive lower GI bleeding	Immunoglobulin (0.5 g/kg), steroids (10 mg/m^2^), etoposide (150 mg/m^2^), cyclosporine (6 mg/kg), and recombinant factor VIIa (rFVIIa) 90 *μ*g/kg (total 4 mg). Resection of 12 cm of Ileum	Died
		4	—	Generalized edema and massive GI bleeding	Recombinant factor VIIa (rFVIIa) 90	Died

MOF: multiorgan failure.
